# Supplementary material for: Mechanisms for the prevention of adolescent intimate partner violence: A realist review of interventions in low- and middle-income countries
Source: PLOS Glob Public Health. 2022 Nov 2;2(11):e0001230. doi: 10.1371/journal.pgph.0001230 (PMC10022317; doi:10.1371/journal.pgph.0001230)
Supplement: S1 Table — (DOCX) [file pgph.0001230.s001.docx]

**Supporting information 1: Search strategy**

| **Number** | **Concept** | **Terms** | **Searches** |
| --- | --- | --- | --- |
| **1** | Adolescent | adolescent* OR teen* OR young people OR youth* OR school age* | *Mapped to MeSH terms with tiab restriction where appropriate.*  To retrieve quantitative evaluations: (1 and 2 and 3 and 4 and 5)  To retrieve qualitative evaluations (1 and 2 and 3 and 4 and 5 and 6) |
| **2** | Intimate Partner Violence (IPV) | IPV OR intimate partner violence OR domestic Violence OR domestic abuse OR partner violence OR partner abuse OR dating violence OR relationship violence OR ADV OR gender-based violence OR GBV OR sexual violence |  |
| **3** | Low- and middle-income country (LMIC) | Afghanistan or Albania or Algeria or Angola or Antigua or Barbuda or Argentina or Armenia or Armenian or Aruba or Azerbaijan or Bahrain or Bangladesh or Barbados or Benin or Byelarus or Byelorussian or Belarus or Belorussian or Belorussia or Belize or Bhutan or Bolivia or Bosnia or Herzegovina or Hercegovina or Botswana or Brasil or Brazil of Bulgaria or Burkina Faso or Burkina Fasso or Upper Volta or Burundi or Cambodia or Khmer Republic or Kampuchea or Cameroon* or Cameron or Camerons or Cape Verde or Central African Republic or Chad or Chile or China or Colombia or Comoros or Comoro Islands or Comores or Mayotte or Congo or Zaire or Costa Rica or Cote dIvoire or Ivory Coast or Croatia or Cuba or Cyprus or Czechoslovakia or Czech Republic or Slovakia or Slovak Republic or Djibouti or French Somaliland or Dominica or Dominican Republic or East Timor or East Timur or Timor Leste or Ecuador or Egypt or United Arab Republic or El Salvador or Eritrea or Estonia or Ethiopia or Fiji or Gabon or Gabonese Republic or Gambia or Gaza or Georgia Republic or Georgian Republic or Ghana or Gold Coast or Greece or Grenada or Guatemala or Guinea or Guam or Guiana or Guyana or Haiti or Honduras or Hungary or India or Maldives or Indonesia or Iran or Iraq or Isle of Man or Jamaica or Jordan or Kazhakstan or Kazakh or Kenya or Kiribati or Korea or Kosovo or Kyrgystan or Kirghizia or Kyrgyz Republic or Kirghiz or Kirgizstan or Lao PDR or Laos or Latvia or Lebanon or Lesotho or Batusoland or Liberia or Libya or Lithuania or Macedonia or Madagascar or Malagasy Republic or Malaysia or Malaya or Malay or Sabah or Sarawak or Malawi or Nyasaland or Mali or Malta or Marshall Islands or Mauritania or Mauritius or Agalega Islands or Mexico or Micronesia or Middle East or Moldova or Moldovia or Moldovian or Mongolia or Montenegro or Morocco or Ifni or Mozambique or Muanmar or Myanma or Burma or Namibia or Nepal or Netherlands Antilles or New Caledonia or Nicaragua or Niger or Nigeria or Northern Mariana Islands or Oman or Muscat or Pakistan or Palau or Palestine of Panama or Paraguay or Peru or Philippines or Philipines or Phillipines or Phillippines or Poland or Portugal or Puerto Rico or Romania or Rumania or Roumania or Russia or Russian or Rwanda or Ruanda or Saint Kitts or St Kitts or Nevis or Saint Lucia or St Lucia or Saint Vincent or St Vincent or Grenadines or Samoa or Samoan Islands or Navigator Island or Navigator Islands or Sao Tome or Saudi Arabia or Senegal or Serbia or Montenegro or Seychelles or Sierra Leone or Slovenia or Sri Lanka or Ceylon or Solomon Islands or Somalia or South Africa or Sudan or Suriname or Surinam or Swaziland or Samoa or Syria or Tajikistan or Tadzhikistan or Tadjikistan or Tadzhik or Tanzania or Thailand or Togo or Togolese Republic or Tonga or Trinidad or Tobago or Tunisia or Turkey or Turkmenistan or Turkmen or Uganda or Ukraine or Uruguay or USSR or Soviet Union or Union of Soviet Socialist Republics or Uzbekistan or Uzbek or Vanuatu or New Hebrides or Venezuela or Vietnam or Viet Nam or West Bank or Yemen or Yugoslavia or Zambia or Zimbabwe or Rhodesia or developing countr* or less* developed countr* or under developed countr* or underdeveloped countr* or middle income countr* or low* income countr* lmic or lmics or low income countr* or middle income countr* or low and middle income countr* |  |
| **4** | Prevention | Prevent* or promot* |  |
| **5** | Intervention | Intervention* OR program* OR evaluat* OR quasi-experiment* or experiment* or RCT or controlled trial or effectiveness or effect* or descrease* or improve* or reduc* or comparative study |  |
| **6** | Qualitative | Qualitative OR ethnograph* OR case stud* OR in-depth interview* OR focus-group discussion* OR interview OR thematic OR grounded theory OR phenomenological |  |
