# Supplementary material for: Mechanisms for the prevention of adolescent intimate partner violence: A realist review of interventions in low- and middle-income countries
Source: PLOS Glob Public Health. 2022 Nov 2;2(11):e0001230. doi: 10.1371/journal.pgph.0001230 (PMC10022317; doi:10.1371/journal.pgph.0001230)
Supplement: S3 Table — (DOCX) [file pgph.0001230.s003.docx]

|  | | | | | **Study details** | | | | | | | |
| --- | --- | --- | --- | --- | --- | --- | --- | --- | --- | --- | --- | --- |
| **Number** | **Person Extracting** | **Publication type** | **Publication year** | **Intervention name** | **Geographic Area** | **IPV definition** | **Target population** | **Target age** | **Sampling** | **Sample characteristics** | **Sample size** | **Type of study** |

**Supporting Information 3: Data extraction table headings and subheadings**

| **Intervention** | | | | | | | | | | |
| --- | --- | --- | --- | --- | --- | --- | --- | --- | --- | --- |
| **Aim** | **Setting** (e.g. school, community etc.) | **Type** (e.g. education, skills training, communication campaign etc.) | **Details/ Activities/ Content** | **Delivery mode** | **Duration/ frequency** | **Mechanisms**  *(theoretical or hypothesised in the intro/ methods/ discussion, or presented as evidence in the results)* | **Adaptations** *(from original intervention or during this intervention)* | **Description of staff that delivered the intervention** | **Staff training/ supervision** | **Staff reflections** |

| **Outcomes** | | | |  | | | |  | | | | |
| --- | --- | --- | --- | --- | --- | --- | --- | --- | --- | --- | --- | --- |
| **Definitions** | **Measures** | **Time frame of measures** | **Findings** *(including adverse effects)* | **Other contextual notes** | **Other mechanism notes** | **Authors' reflections** | **Conclusions** | **Notes/Unsure items** | **Reference** | **Link** | **Review paper** | **Related materials** |
