# Supplementary material for: Mechanisms for the prevention of adolescent intimate partner violence: A realist review of interventions in low- and middle-income countries
Source: PLOS Glob Public Health. 2022 Nov 2;2(11):e0001230. doi: 10.1371/journal.pgph.0001230 (PMC10022317; doi:10.1371/journal.pgph.0001230)
Supplement: S4 Table — (DOCX) [file pgph.0001230.s004.docx]

**Supporting information 4: Dataset**

Data supporting the results presented in this article are drawn from open access published data. See list below.

| **Reference number in manuscript** | **Reference** | **Link** |
| --- | --- | --- |
| 32 | Makleff S, Garduño J, Zavala RI, Barindelli F, Valades J, Billowitz M, et al. Preventing Intimate Partner Violence Among Young People—a Qualitative Study Examining the Role of Comprehensive Sexuality Education. Sex Res Soc Policy. 2020 Jun 1;17(2):314–25. | <https://link.springer.com/article/10.1007/s13178-019-00389-x> |
| 33 | Jewkes R, Nduna M, Levin J, Jama N, Dunkle K, Puren A, et al. Impact of Stepping Stones on incidence of HIV and HSV-2 and sexual behaviour in rural South Africa: cluster randomised controlled trial. BMJ. 2008 Aug 7;337:a506. | <https://pubmed.ncbi.nlm.nih.gov/18687720/> |
| 34 | Mathews C, Eggers SM, Townsend L, Aarø LE, de Vries PJ, Mason-Jones AJ, et al. Effects of PREPARE, a Multi-component, School-Based HIV and Intimate Partner Violence (IPV) Prevention Programme on Adolescent Sexual Risk Behaviour and IPV: Cluster Randomised Controlled Trial. AIDS Behav. 2016 Sep;20(9):1821–40. | <https://pubmed.ncbi.nlm.nih.gov/27142057/> |
| 35 | Kågesten AE, Oware PM, Ntinyari W, Langat N, Mboya B, Ekström AM. Young People’s Experiences With an Empowerment-Based Behavior Change Intervention to Prevent Sexual Violence in Nairobi Informal Settlements: A Qualitative Study. Glob Health Sci Pract. 2021;9(3):508–22. | <https://www.ncbi.nlm.nih.gov/pmc/articles/PMC8514032/> |
| 36 | Pulerwitz J, Hughes L, Mehta M, Kidanu A, Verani F, Tewolde S. Changing Gender Norms and Reducing Intimate Partner Violence: Results From a Quasi-Experimental Intervention Study With Young Men in Ethiopia. Am J Public Health. 2015 Jan;105(1):132–7. | <https://www.ncbi.nlm.nih.gov/pmc/articles/PMC8514032/> |
| 37 | Verma R, Pulerwitz J, Mahendra V, Khandekar S, Singh AK, Das SS, et al. Promoting gender equity as a strategy to reduce HIV risk and gender-based violence among young men in India. HIV AIDS [Internet]. 2008 Jan 1; Available from: https://knowledgecommons.popcouncil.org/departments_sbsr-hiv/31 | <https://knowledgecommons.popcouncil.org/departments_sbsr-hiv/31/> |
| 38 | Sosa-Rubi SG, Saavedra-Avendano B, Piras C, Van Buren SJ, Bautista-Arredondo S. True Love: Effectiveness of a School-Based Program to Reduce Dating Violence Among Adolescents in Mexico City. Prev Sci. 2017 Oct 1;18(7):804–17. | <https://link.springer.com/article/10.1007/s11121-016-0718-4> |
| 39 | Naved RT, Mamun MA, Mourin SA, Parvin K. A cluster randomized controlled trial to assess the impact of SAFE on spousal violence against women and girls in slums of Dhaka, Bangladesh. PLOS ONE. 2018 Jun 14;13(6):e0198926. | <https://journals.plos.org/plosone/article?id=10.1371/journal.pone.0198926> |
| 42 | Jewkes R, Gevers A, Chirwa E, Mahlangu P, Shamu S, Shai N, et al. RCT evaluation of Skhokho: A holistic school intervention to prevent gender-based violence among South African Grade 8s. PLOS ONE. 2019 Oct 29;14(10):e0223562. | <https://journals.plos.org/plosone/article?id=10.1371/journal.pone.0223562> |
| 43 | Decker MR, Wood SN, Ndinda E, Yenokyan G, Sinclair J, Maksud N, et al. Sexual violence among adolescent girls and young women in Malawi: a cluster-randomized controlled implementation trial of empowerment self-defense training. BMC Public Health. 2018 Dec;18(1):1341. | <https://bmcpublichealth.biomedcentral.com/articles/10.1186/s12889-018-6220-0> |
| 44 | Kilburn KN, Pettifor A, Edwards JK, Selin A, Twine R, MacPhail C, et al. Conditional cash transfers and the reduction in partner violence for young women: an investigation of causal pathways using evidence from a randomized experiment in South Africa (HPTN 068). J Int AIDS Soc. 2018 Feb 27;21(Suppl 1):e25043. | <https://pubmed.ncbi.nlm.nih.gov/29485746/> |
| 45 | Sinclair J, Sinclair L, Otieno E, Mulinge M, Kapphahn C, Golden NH. A Self-Defense Program Reduces the Incidence of Sexual Assault in Kenyan Adolescent Girls. J Adolesc Health. 2013 Sep;53(3):374–80. | <https://pubmed.ncbi.nlm.nih.gov/23727500/> |
| 46 | Jewkes R, Wood K, Duvvury N. ‘I woke up after I joined Stepping Stones’: meanings of an HIV behavioural intervention in rural South African young people’s lives. Health Educ Res. 2010 Dec 1;25(6):1074–84. | <https://www.ncbi.nlm.nih.gov/pmc/articles/PMC3003491/> |
| 47 | MacPhail C, Khoza N, Selin A, Julien A, Twine R, Wagner RG, et al. Cash transfers for HIV prevention: what do young women spend it on? Mixed methods findings from HPTN 068. BMC Public Health. 2017 Jul 11;18(1):10. | <https://pubmed.ncbi.nlm.nih.gov/28697762/> |
| 48 | Makleff S, Garduño J, Zavala RI, Valades J, Barindelli F, Cruz M, et al. Evaluating Complex Interventions Using Qualitative Longitudinal Research: A Case Study of Understanding Pathways to Violence Prevention. Qual Health Res. 2021 Jul 1;31(9):1724–37. | <https://journals.sagepub.com/doi/full/10.1177/10497323211002146> |
| 49 | Makleff S, Billowitz M, Garduño J, Cruz M, Silva Márquez VI, Marston C. Applying a complex adaptive systems approach to the evaluation of a school-based intervention for intimate partner violence prevention in Mexico. Health Policy Plan. 2020 Oct 1;35(8):993–1002. | <https://pubmed.ncbi.nlm.nih.gov/32761146/> |
