# Supplementary material for: Mechanisms for the prevention of adolescent intimate partner violence: A realist review of interventions in low- and middle-income countries
Source: PLOS Glob Public Health. 2022 Nov 2;2(11):e0001230. doi: 10.1371/journal.pgph.0001230 (PMC10022317; doi:10.1371/journal.pgph.0001230)
Supplement: Attachment — Submitted filename: Response to reviewers_final.docx [file pgph.0001230.s005.docx]

**Response to reviewers**

We would like to thank the three reviewers for their insightful comments on the manuscript. We believe these revisions have strengthened our paper and hope the reviewers agree and find the changes satisfactory. We provide a detailed response to each of these comments in the table below.

| **Comment** | **Response** |
| --- | --- |
| Reviewer 1 | |
| A careful spell check throughout manuscript is required e.g. RAMSES instead of RAMESES | Thank you for noticing this typo. We have amended the text on line 110 of page 6. We have thoroughly spell checked the entire manuscript. |
| The authors are not including adverse effects in their programme theory - considering the nature of the topic it would be good to explain how this was or was not investigated, and why | Whilst we explored both positive and adverse intervention effects, adverse effects were rarely reported. When they were reported, they were not thought to be related to the intervention. As such, adverse effects were not included in the model. |
| Whilst it is fully understood that the realist review method is complex and it is difficult to be fully transparent about how the programme theory was derived from the data, it would be helpful if authors could share the full data extraction tables (authors mention they used a Microsoft Excel document and it might be helpful to share this) | Thank you for this suggestion. We have included the data extraction table in the supplementary material (Supplementary Appendix 3). |
| Whilst authors explain some of the limitations of the review method, there is not much information about limitations with regards to applying the realist review method to developing the programme theory; in particular what were challenges around including findings of studies that did not provide sufficient detail for the purpose of informing a programme theory; was the relevance of studies assessed? | While we touch on this point briefly on page 33 in relation to a lack of qualitative evaluations which theorise about the mechanisms of change, we agree that this was a particular challenge specific to the realist methodology and have included a sentence to expand on this at the end of paragraph one on page 33:  “As such, analysing the data presented in these studies using a realist framework was challenging and we often had to draw upon wider literature on similar interventions in different populations to explore the potential mechanisms.”  We reviewed the relevance of studies throughout the screening process to determine if they were likely to make a contribution to the development, refinement and testing of theory. While some evaluations lacked in-depth exploration of potential mechanisms, we believed all included studies at least contributed to the initial development of theory, even if we had to turn to broader literature for refinement and testing. |
| Reviewer 2 | |
| There is a minor linguistic edit required to the piece. | We have thoroughly read through the manuscript to ensure appropriate and concise language is used. |
| Additionally the piece would benefit more from a clearer methods/analysis section. Though the data is adequately presented, the paper lacks a bit of a walkthrough to how these results were achieved. | We have used a 4-stage process that takes the reader through what we did and have tried to be as explicit and transparent as possible about the data analysis process. The process was iterative and a collaboration between 7 authors, which we appreciate might be difficult to follow because the nature of conducting a realist review which does not have a clear set of distinct steps. The final model was the product of many lengthy conversations between the research team. We have added extra details to the methods in the ‘Stage 4’ (page 9-10) section to give specific examples of how the model was refined. |
| The paper would also benefit from a stronger wrap-up section, bringing the discussion back to why IPV is in fact an 'adolescent' issue through an intersectional lens. Why it is pivotal for IPV among adolescents to be separated from larger work on GBV and IPV among adults, and how impeding factors such as conflict, poverty, displacement and social norms frame this. A more elaborate and comprehensive conclusion will lay the foundation for further research, and adequately frame findings within the necessary intersectional realities. | Thank you for this valuable suggestion. We have edited the conclusion on page 34 to provide a stronger final message. |
| Reviewer 3 | |
| Page 13; Para 2: The authors argue that follow-up after 6 months might not be appropriate for observing the sustainability of impact. What can be the minimal duration of follow-up for observing the impact? | It may be possible that 6 months is an appropriate duration to observe impact, but we would need a longer duration of follow up to make this claim. Similarly, a 6-month duration cannot tell us if the intervention had a sustained impact on the outcomes we are exploring, as we state on page 13. While the appropriate duration of follow-up is highly dependent on the intermediary mechanisms and outcomes the interventions are aiming to achieve, we can see from this review that those interventions that had longer follow-up periods and collected longitudinal data throughout this period were more able to explore in-depth the potential mechanisms of change. |
| Page 21-27: The findings and discussion might have intermixed here. Some content from this section can be moved to discussion section. | In this review, as is typical of realist reviews, we drew upon broader literature as part of the analysis process. This literature was used to refine our programme theory and because of this, we have included it as evidence in the results section, mostly towards the ends of the paragraphs as a means of providing evidence for that mechanism. We appreciate this is not typical of other review methodologies, but believe it is essential for a realist review in order to provide evidence for our proposed mechanisms. We have however removed any references to other literature where they are not being used as evidence to support our theoretical model. |
| Page 26 para 1 and Figure 2: The authors have identified peer pressure was effective in reduction in perpetration and experience of IPV among adolescents. However, several meta-analyses in the past have reported that peer education have limited effects in promoting healthy behaviours and improving health outcomes among target groups. Those evidence have reported that peer education programs mainly benefit peer educators rather than their intended beneficiaries. Can the authors discuss about this? | Thank you for this comment. We agree with the point being made but to clarify, we have not identified peer pressure as effective in reducing IPV perpetration and experience. Rather we identify peer groups as safe spaces in which adolescents can participate in interventions. Our analyses suggest, as illustrated in figure 2, that it is this mode of intervention delivery that supports the main mechanism of change which is the reconceptualization of gender norms and beliefs. The mechanism is the safe space that the peer groups provide, rather than the peer pressure element of peer groups. In fact, peer pressure did not come up as a contributing factor to the success of interventions in this review and as such we do not discuss it.  Regarding peer education programmes, the interventions in this review that used peer facilitators were not peer education programmes. They were different types of interventions that sometimes used a facilitator who had similar characteristics to those of the participants. |
| Page 11, Line 216: The countries should be 7. Please explain if any of this is not LMIC. | Thank you for noticing this. It should be 7 countries and they are all LMICs. We have amended the text on page 11 line 219. |
| Page 11: Line 235: ‘Nearly all’ does not add the value here. | We have changed the wording of this sentence on page 11 line 237 to “The majority of interventions (n=9)…”. |
| Page 11, Line 237: Reference Typo (32-39)? | This was a typo and has been corrected. |
| Page 13: Line 290: Reduction in experience of sexual assault among the intervention group after self-defence training for girls was from Kenya while Malawi study did not show any reduction in sexual assault by boyfriends as per Table -1: Please check it. | This is correct, it is the intervention in Kenya which saw a reduction in sexual assault by boyfriends. We have amended the text on page 13 line 292 to reflect this:  “The self-defence empowerment training for girls in Kenya found a reduction in the experience of sexual assault among the intervention group, but no reductions in sexual assault by a boyfriend (45).” |
| Page 23, Para 1: The definition of Adolescents might be appropriate in Background section rather than in the findings section. Some sentences might require references here. | Thank you for this suggestion. As this definition is already in the background section, we have removed it from the findings. |
